# The Phenomenology of Remembering Is an Epistemic Feeling

**DOI:** 10.3389/fpsyg.2020.01531

**Published:** 2020-07-03

**Authors:** Denis Perrin, Kourken Michaelian, André Sant’Anna

**Affiliations:** Institute of Philosophy in Grenoble, Centre for Philosophy of Memory, Université Grenoble Alpes, Grenoble, France

**Keywords:** episodic remembering, phenomenology, autonoesis, metacognition, epistemic feelings, feeling of pastness

## Abstract

This article aims to provide a psychologically informed philosophical account of the phenomenology of episodic remembering. The literature on epistemic or metacognitive feelings has grown considerably in recent years, and there are persuasive reasons, both conceptual and empirical, in favor of the view that the phenomenology of remembering—*autonoetic consciousness*, as Tulving influentially referred to it, or the *feeling of pastness*, as we will refer to it here—is an epistemic feeling, but few philosophical treatments of this phenomenology as an epistemic feeling have so far been proposed. Building on insights from the psychological literature, we argue that a form of feeling-based metacognition is involved in episodic remembering and develop an integrated metacognitive feeling-based view that addresses several key aspects of the feeling of pastness, namely, its status as a feeling, its content, and its relationship to the first-order memories the phenomenology of which it provides.

## Introduction

There is a broad consensus, in both psychology and philosophy, that episodic remembering is characterized by a specific phenomenology. Tulving (e.g., 1985), the most influential proponent of this view in psychology, argued that what it is to episodically remember is to have a mental image of a past event accompanied by the consciousness—*autonoesis*, in his terms—of mentally traveling back to one’s original experience of that event. In episodic memory, one does not, in other words, merely *know* that the represented event occurred in one’s past; one in some sense *relives it:* to episodically remember is “to consciously re-experience past experiences” (Tulving, 2002b: 6). As Klein puts it, in a Tulvingian vein, if awareness of content is to amount to remembering, “the content in awareness must present itself as a reexperience of an experience previously had” Klein (2015a: 6). From a philosophical point of view, the feature of episodic remembering that Tulving and Klein single out is its qualitative or phenomenal character—its *what-it-is-likeness*, to use an intuitive but vague expression present in the literature. It is the nature of this what-it-is-likeness that the present article aims to describe and explain. Before proceeding, we note that there are three related issues with which the article will not be concerned. First, though doubts have occasionally been voiced (e.g., by Hoerl, 2019) regarding the very existence of a specific phenomenology of remembering, the article will not respond to these doubts, taking the existence of this feature of remembering to be both intuitively plausible and empirically well-established. Second, it will not consider the question whether, in order for a mental state to qualify as an episodic memory, it must involve a specific phenomenology; that is, it will not argue that the latter is a *necessary*—and not merely a characteristic—feature of episodic remembering (compare Martin and Deutscher, 1966; Wheeler et al., 1997). Finally, the article will not be concerned with the question whether the phenomenology of remembering is *sufficient* for episodic remembering: certain “continuist” (as opposed to “discontinuist”; see Perrin and Michaelian, 2017; Michaelian et al., 2020) approaches to the relationship between episodic memory, on the one hand, and episodic future and counterfactual thought, on the other hand, have treated the latter as being characterized by a phenomenology that is, modulo its temporal orientation, identical to that of the former (e.g., Tulving, 2002b; De Brigard, 2014; Michaelian, 2016), but we will remain agnostic on this question here, contenting ourselves with giving an account of the phenomenology of episodic memory without considering its relationship to the phenomenologies of other forms of episodic thought.

This article aims to provide a psychologically informed philosophical account of the phenomenology of episodic remembering. The literature on epistemic or metacognitive feelings has grown considerably in recent years, and there are persuasive reasons, both conceptual and empirical, in favor of the view that the phenomenology of remembering—the *feeling of pastness* (FOP), as we will refer to it here^1^ —is an epistemic feeling, but few philosophical treatments of this phenomenology as an epistemic feeling have so far been proposed. Building on insights from the psychological literature, we argue that a form of feeling-based metacognition is involved in remembering and develop an integrated metacognitive feeling-based view that addresses several key aspects of the feeling of pastness, namely, its status as a feeling, its content, and its relationship to the first-order memories the phenomenology of which it provides. We proceed as follows. In section “Introducing the Feeling View of Episodic Phenomenology,” we introduce the view on which the phenomenology of remembering is a feeling and that its content is not limited to pastness as such but also involves components pertaining to the self, causality, and singularity. In section “Feeling-Phenomenology Without Metacognition,” we discuss non-metacognitive accounts of the feeling phenomenology of remembering. Finding these to be unsatisfactory, we turn in section “Dokic’s Metacognitive Feeling-Based Account” to the only metacognitive feeling-based account of the FOP currently available, that proposed by Dokic (2014), but argue it faces a number of serious problems. Building on the critical discussion of Dokic’s proposal, we develop, in section “A New Metacognitive Feeling-Based Account,” a new metacognitive feeling-based account. This account incorporates a moderate anti-separatist position regarding the relationship between the FOP and first-order memories, an inferentialist and embodied position regarding the phenomenal quality of the FOP, and a moderate conceptualist position regarding the content of the FOP, and thereby avoids the problems that beset Dokic’s account.

## Introducing the Feeling View of Episodic Phenomenology

In this section we introduce the view that the phenomenology of remembering is appropriately conceived of as a feeling in the first place, and ask which components the content of such a feeling includes. Once we will have secured these basic points of characterization, we can embark upon the search for the feeling account to prefer in the following sections.

### Is the Phenomenology of Remembering a Feeling?

Two main types of accounts are proposed in the existing literature about the phenomenology of episodic remembering. Some hold that it consists of a judgment or meta-comment made about the information conveyed by a first-order memory. On the so-called meta-representationalist stand^2^, what is represented by a first-order memory appears *as past* because a comment built into the content of this memory states it is so. Accounts of the latter sort have recently been proposed by Redshaw (2014), Mahr and Csibra (2018). Redshaw (2014) refers to the phenomenology of remembering in terms of autonoesis and claims it requires the capacity to relate one’s memories to other aspects of reality by situating first-order episodic representations with respect to larger narrative structures. For instance, a subject’s first-order representation of his tenth birthday party will only involve autonoesis, on this account, if the subject is able to place it in a more general narrative of his past, that is, if he can locate this event in relation to other events experienced by him. The ability to undergo autonoesis is thus, if Redshaw is right, bound up with the ability to metarepresent relationships among different first-order episodic representations. In a broadly similar vein, Mahr and Csibra argue that memory serves a communicative function in that it enables the subject to establish himself as a reliable communicator, with autonoesis serving to flag the relationship between memories and past experiences, thereby enabling the subject to claim epistemic authority with respect to remembered events. For instance, if the subject wants to convince his friends that the mayor punched a citizen, it will be beneficial for him if his memory of that event is accompanied by a state signaling that the memory originates in his experience of the remembered event. The ability to undergo autonoesis is thus, if Mahr and Csibra are right, bound up with the ability to metapresent the relationship between present memories and past experiences.

Others are eager to describe the phenomenology of episodic remembering in terms of feeling and attempt to explain it by appealing to the informational content of the first-order memory representation itself. Fernández (2006, 2017), for instance, develops an account according to which the temporal phenomenology of episodic remembering consists of the feeling of pastness, which he defines as the feeling that what a memory represents occurred in the past. On his view, this phenomenology must be explained in terms of the experience of the content of the memory it accompanies. Specifically, we would undergo a feeling of pastness because the content of the memory says about itself that it causally derives from a personal experience. We will have more to say about this experience-of-content account in section “Feeling-Phenomenology Without Metacognition.” For now, we point out that regardless of their differences, these accounts all posit a strong dependence relation between the phenomenology of the episodic remembering and the content of the first-order memory whose phenomenology it is. On the meta-representationalist account, the phenomenology is a judgment whose object is the content of the first-order memory, thus the content of the judgment includes the latter. On the experience-of-content account, the phenomenology is an experience whose object is the content of the first-order memory, thus the phenomenology is strongly determined by the content of the memory. While we will argue for an account of the phenomenology of episodic remembering in terms of feelings rather than in terms of metarepresentational judgments, we think that these feelings are largely not dependent on the content of the first-order memory (vs. the experience-of-content account) but produced metacognitively, as claimed by Dokic’ account in terms of feeling-based metacognition^3^.

### What Are the Components of the Feeling?

We will, in what follows, assume that the phenomenology of remembering consists of a feeling, whether or not it is properly classified as a metacognitive feeling. Now what is the content of this feeling? There are three obvious components that the content might include. First, it might inform the subject that the remembered event occurred in the past; call this the *pastness* component. Second, it might inform him that it occurred in *his* past—that he experienced it when it happened; call this the *self* component. And third, it might inform him that his current representation of the remembered event results from his past experience of it; call this the *causation* component. While there is no uncontroversial means of determining the content of a given phenomenal state, and while atypical cases have elicited and will certainly continue to elicit controversy, it is plausible that the phenomenology of remembering includes each of these three components. Our argument, however, does not require us to make the strong claim that each of the components is *necessary* for it;^4^ it is enough, for present purposes, to maintain that they are *typical* of that phenomenal state. Thus it may be, for all that we say here, that, in certain atypical cases, the phenomenology of remembering does not include all three components. This, in turn, has the consequence that, even if we grant that a specific phenomenology is necessary for the occurrence of genuine episodic remembering, there may be cases of genuine episodic remembering—this is what suggests the case of RB described by Klein and Nichols (2012)—in which, for example, the subject does not have the sense that the remembered event belongs to his past or that he experienced it when it occurred.

Why focus on these three components? The pastness component is uncontroversial. Though Tulving (2002a) does argue in places that the sense of time involved in episodic memory, *chronesthesia*, can be separated from the sense of self, autonoesis, he sees both chronesthesia and autonoesis as playing essential roles in remembering. In practice, moreover, the difference between the concept of autonoesis and that of chronesthesia is typically treated as being a matter of mere emphasis: as Szpunar puts it, “[t]he concept of ‘chronesthesia’ emphasizes awareness of the subjective time in which one’s self exists,” whereas “[t]he concept of ‘autonoetic consciousness’ emphasizes awareness of one’s self existing in subjective time” Szpunar (2011: 410). The self component is likewise basically uncontroversial. The central role of a sense of the self lies, for example, behind Tulving’s and Szpunar’s distinction between autonoesis and chronesthesia. And the case described by Klein and Nichols would be uninteresting were we not to take it that the phenomenology of remembering at least typically involves a sense of the self: the case of patient RB is provocative precisely because it seems to be one in which every other characteristic that we ordinarily attribute to episodic memory—both at the level of content (RB’s representations of past events include detailed sensory information about particular events) and at the level of phenomenology (he is aware that the events happened in the past and that his current representations of them are caused by his past experience of them)—is present but in which the subject lacks the sense that the events that he remembers belong to him. While Klein and Nichols’ description of this particular case has been contested by some (Roache, 2016; see Klein, 2016 for a reply), others (Fernández, 2019) have taken it as the basis for arguments for the involvement of a feeling of ownership in episodic memory, and this general claim is plausible regardless of what we make of the case of RB in particular.

The causality component may be more controversial. We point out, however, that the involvement in the phenomenology of remembering of a sense that one’s present representation causally originates in one’s past experience of the represented event would account for the enduring popularity of the causal theory of memory (Martin and Deutscher, 1966; Bernecker, 2010; Michaelian and Robins, 2018). Consider the example offered by Dokic (2014) of his memory of flying on the Concorde as a young child, a memory which, he tells us, traces back, not to his experience of flying on the Concorde, but rather to his parents’ telling him about his flying on the Concorde several years after the event. The intuition that such memories are in an important sense defective—that they are, as philosophers sometimes say, *merely apparent*, as opposed to *genuine*—is plausibly accounted for by the fact that, when one remembers, one has the sense that one’s memory is due to one’s experience of the remembered event. Thus even Fernández (2019), who rejects the causal theory, builds a representation of a causal link between the present representation and the past experience into the content of the memory in an attempt to account for the phenomenology of remembering. Similarly, Mahr and Csibra (2018, 2020), we have seen, argue that memory is able to perform its communicative function because its phenomenology indicates to the rememberer the existence of a causal relationship between his memory and his experience of the corresponding event—episodic memory is able to serve as a “witness trump card” (Henry and Craver, 2018) because its phenomenology includes causality (“I remember this because I saw it happen”), as opposed to mere actuality (“This happened”). Accordingly, we will take it for granted, in what follows, that the phenomenology of remembering includes the causation component, as well as the pastness and self components.

In addition to these three components, the inclusion of which in the phenomenology of remembering is relatively widely recognized, phenomenology includes, we want to suggest, a fourth component: singularity (Perrin, 2019; Sant’Anna, 2020)^5^. In line with the literature on singular thought (Jeshion, 2010), by singularity we mean a quantity feature. As far as episodic memory is concerned, it characterizes the particularity of experienced and remembered events that are represented by the memories, for instance such or such particular event of going to the university on a specific day (e.g., the time I meet an old friend on the tram as I was heading toward the university), in contrast with the iterative event of doing so. On standard accounts of the content of episodic memories, they typically represent singular events in this sense. So, it seems important to include singularity among the components of the content of episodic memory. The content of many episodic memories is formed of an imagistic content plus a certain phenomenology. Now imagistic contents do not include by themselves any quantificational feature of the represented event. Our suggestion is that the singularity feature of remembered events can be conveyed by phenomenology. One could object that it is an additional semantic information that fulfills this role. But we experience episodic memories in which the represented event appears as having occurred at a singular moment of our past experience while no semantic information is accessible regarding this quantificational feature and we are unable to assign a specific spatio-temporal address. Thus, our proposal is that the information that the remembered content is singular can be conveyed by phenomenology and is one of its typical components. We will elaborate on this in section “A New Metacognitive Feeling-Based Account.” If the phenomenology of remembering includes these four components—pastness, self, causality, and singularity—how, exactly, are they related to each other? We take this question up in the section “The Content of the FOP: Moderate Conceptualism.”

## Feeling-Phenomenology Without Metacognition

In the previous section, we introduced an approach to the phenomenology of remembering as a feeling and we detailed its typical components. The question that now confronts us is whether the mechanism that gives rise to this phenomenology is metacognitive in nature or not. In this section, we review and reject two views—defended, respectively, by Klein and Fernández—that attempt to account for the phenomenology of remembering in terms of feelings but without invoking underlying metacognitive mechanisms^6^. Such views turning out to be inadequate, we will consider the metacognitive feeling-based approach in the following section.

### The Structural Approach

On the Tulving-inspired (Tulving, 1985; Wheeler et al., 1997: 350) approach to episodic memory defended by Klein, for a mental state that is causally connected to past experience to qualify as an episodic memory it is necessary and sufficient that it be accompanied by autonoetic consciousness, where autonoesis is understood as a feeling. Klein speaks, for instance, of a “subjective feeling of pastness” Klein (2015a: 5), i.e., of a phenomenal character of remembering that is distinct from, but associated with the content of retrieved memories: “‘there is something it is like’ for a mental state to be experienced as an act of remembering” (2015a: 1). Autonoetic consciousness, on Klein’s view, “is a *direct, non-inferential feeling given to awareness*” (2015a: 8, emphasis added) and includes the four components identified in section “Feeling-Phenomenology Without Metacognition:” contrasting autonoetic consciousness with the noetic consciousness involved in semantic remembering, for example, Klein argues that “noetic consciousness does not provide its owner with a subjective feeling that she or he is mentally traveling back in time [pastness] to the events and experiences [singularity] that gave birth to [causality] the content in awareness. … the feeling of subjective time travel [self] is intrinsic to autonoesis” Klein (2015b: 5).

While we are in agreement with Klein regarding the components of autonoesis, we find his account to be problematic in other respects. Borrowing a term from Whittlesea (2003: 241), Klein’s approach proposes a “structural account” insofar as “it assumes that each qualitatively different aspect of performance is supported by a different module of memory.” In other words, Klein conceives of autonoetic consciousness as a distinct and autonomous structural component of the mind rather than as the outcome of the working of a certain mechanism. This approach is inadequate in two respects. First, it has nothing to say about how the components of the content of autonoesis relate to each other. Second, it dispenses with providing an account of the processes that bring about autonoetic consciousness while there are serious potential candidates to this role. Klein thus suggests that autonoesis is a self-contained and ready-made element of the cognitive system, which rules out accounts that see it as depending on other elements of the system, e.g., on the contents of memories or on the detection and interpretation of features of the processing that produces them. Overall, the account is unsatisfactory in that, first, it builds a good deal of conceptual richness—pastness, self, causality, singularity—into autonoesis without explaining how something that has the nature of a feeling is capable of having such a conceptually rich content; and second, in that it excludes the possibility that autonoesis be generated by certain mechanisms in the cognitive system, and subsequently does not really propose an account of autonoesis.

### The Experience-of-Content Approach

Unlike Klein, Fernández does propose an explanation of the basis of the phenomenology of episodic recollection. He appeals to an intentionalist approach, according to which the phenomenology of a mental state is accounted for in terms of its representational content (Dretske, 1997; Tye, 1997, 2002; Byrne, 2001). His specific intentionalist explanation appeals to the contents of retrieved memories rather than on metacognitive monitoring of the processing that produces them: “the content of a memory is responsible for the way in which the memory feels to the subject” (2019: 29). Fernández’ hypothesis is, more precisely, that the phenomenology of remembering is the experience of the content of the memory: “the temporal phenomenology of memory is the way in which we experience some of the things that we represent in memory” (2019: 24).

Fernández argues for a self-referential view of memory content, according to which the content of a memory represents the fact that it (the memory) causally originates in the remembering subject’s earlier experience of the remembered event (2019: 79). In other words, not only the remembered event and the rememberer’s original experience of it but also the notion of a causal relation between the memory and the experience is built into the content of the memory. As he sees things, this explains the occurrence of the pastness component of the content of autonoesis, or what Fernández refers to as “PAST”: awareness of the remembered fact^7^ “as obtaining in the past” (2019: 87)^8^. On his view, PAST is the subject’s experience of the causal component of the content of an episodic memory (2019: 108): because it is intrinsic to the notion of causality that causes precede their effects, when one is aware of the causality component of a memory one is thereby aware that the remembered event is located in the past; experience of the causality component of the memory gives rise to the pastness component of the associated phenomenology (2019: 108−109).

We have three objections to this experience-of-content account. The first is that it is in conflict with Fernandez’ overall first-order representationalist account. The idea that content is experienced and then becomes conscious sounds more like a higher-order theory, such as the ones proposed by Lycan (1996), Rosenthal (1997), who reject first-order representationalism (e.g., Tye’s and Dretske’s views). The second concerns the phrase “experience of some of the things that we represent in memory.” In practice, Fernández has little to say here. If this notion cannot be successfully unpacked, an analysis—such as that proposed in the following section—that succeeds in explaining the occurrence of PAST by providing an empirically grounded description of the mechanism underlying PAST should be preferred. Even if it can be unpacked, there are concerns about the very notion of an experience of the component of a content. In what, precisely, does such an experience consist? We see two possible answers to this question: the experience might be a matter of introspection, or it might be a matter of understanding. Each of these is problematic.

Consider the first option. We might define the relevant notion of experience in terms of introspection, but three problems would then arise. First, if the object of the subject’s introspection is the causality component, then the feeling experienced by the subject as he introspects the causality component should concern causality in addition to pastness. Actually, however, Fernández’ account of PAST leaves no room for consciousness of the causality component. Second, this definition does not explain why introspection of the causality component should trigger a feeling rather than, say, a belief about the temporal location of the remembered fact. In other words, the affective, phenomenal character of PAST is not explained. Finally, it is not clear that something as abstract as the causality component might be the object of introspection—in order to grasp such an entity, understanding would seem to be required.

Consider, then, the second option. We might define the relevant notion of experience in terms of understanding. Fernández’ analysis of intentional content in terms of propositional content suggests this definition, and some neo-Russellian researchers maintain that some of the components of the propositional contents of singular thoughts or sentences are the relata of an experiential relation of acquaintance (Jeshion, 2010). Understanding thoughts of this kind would imply being acquainted with some of the components of their content. But while the idea of an acquaintance relation with a concrete entity is relatively plausible, the idea of such a relation with an abstract entity—such as a causal relation—is obscure at best. In short, there seems to be no satisfactory definition of the experience of a component of content, as it figures in Fernández’ account.

Our third objection requires to introduce some empirical findings that will be pivotal for our argument^9^. There is a great deal of research demonstrating that feelings such as PAST are sensitive to features of the processing that produces mental states, rather than to the content of those states (Whittlesea, 1997: 219; Koriat, 2007: 298; Dokic, 2012: 310). Experiments show that, depending on the questions asked to subjects, one and the same experimental device can trigger PAST as well as (for instance) a feeling of pleasantness. In Whittlesea (1993: 1244−1246) – see also Whittlesea (1997: 246–247), subjects were presented with a list of words in a study phase. In the test phase, they were asked whether the items presented were neutral or pleasant before being asked whether they had been presented with them (“old”) or not (“new”). As it turned out, words presented in the study phase were judged more pleasant than the others, just as they were judged old more often than the others^10^. These similar patterns of results strongly suggest that the output of the detection of one and the same perceptual fluency feature^11^ due to prior presentation can produce a feeling of pleasantness as well as a PAST feeling, depending on the questions asked to the subjects. Now—so our argument goes—these findings strongly threaten Fernández’ analysis. If the occurrence of PAST were sensitive to the content of a memory, Fernández could account in either of two ways for the above results, none of which is satisfactory. Either he could say that the feeling of pleasantness, too, results from the experience of a self-referential causal content. But one would hardly see why pleasantness could be the result of such an experience. Or he could say that two distinct mechanisms are in play as feelings of pleasantness occur and as PAST feelings occur, the former being grounded on fluency-detection while the latter is an experience of the causal self-referential content, but that despite their difference they produce the same patterns of results for the same items. Though not impossible, this option is much more complex and as far as we know not empirically supported. The metacognitive solution suggested above in terms of the detection and interpretation of procedural features thus recommends itself as more plausible than an explanation of PAST in terms of content-sensitivity. However, though Fernandez’ content-based account turns out unsatisfactory, our account claims the plausibility and the sufficiency of the metacognitive approach, and we do not exclude in principle that content could play a role in episodic phenomenology. But arguments for this possibility are still to be provided. In total, therefore, neither of the accounts in terms of non-metacognitive feelings considered reveals satisfactory, and a metacognitive account promises to be more adequate. So, let’s explore this option.

## Dokic’s Metacognitive Feeling-Based Account

In this section, we promote the view in cognitive psychology on which episodic recollection involves epistemic feelings. That provides a good reason to explore as a serious option a feeling-based account of the phenomenology of remembering such as the one favored by Dokic, which we proceed to put under discussion.

### The Metacognitive Stand About the Phenomenology of Remembering

We will argue that the phenomenology of remembering results from metacognitive monitoring. Let us make this claim more precise. We build on views (Koriat and Levy-Sandot, 1999; Proust, 2007; De Sousa, 2009; Arango-Muñoz, 2011), themselves inspired by the dual processing framework (Evans and Stanovich, 2013), that acknowledge the existence of two distinct forms of metacognition. On the one hand, metacognition sometimes takes the form of explicit, controlled, slow, conceptually articulated processing—*type 2* processing, in short—that involves the application of a theory of mind (for which reason it is often seen as being metarepresentational) and consists of conscious (or: personal) processes and outputs^12^. On the other hand, metacognition sometimes takes the form of implicit, automatic, fast, and conceptually poor processing—*type 1* processing—that may (but does not necessarily) produce conscious outputs. Type 1 metacognition relies on heuristics that enable the mind to carry out cognitive operations more rapidly and economically than type 2 metacognition. For instance, if one has to answer a question that taps into one’s knowledge in geography but is unable to do so straightaway, one might decide to keep searching for the answer rather than giving up either because one explicitly remembers that one has studied the relevant material and therefore consciously infers that one should be able to retrieve the answer given sufficient effort, or because one has a gut feeling (the source of which, of course, eventually needs to be explained) that one will be able to retrieve the answer to the question.

The dual processing framework has been applied to memory in a similar way. On the standard application of this framework (see Yonelinas, 2002 for a review), recognition memory can take either of two forms^13^. Consider a standard recognition task in which a study phase during which subjects are presented with a list of words is followed by a test phase during which they have to decide whether items are “old” or “new.” Subjects may, a proponent of the dual processing approach argues, draw either on a feeling of familiarity or on explicit recollection in order to make this decision. The former strategy involves the application of a heuristic, while the latter relies on a detailed and explicit mental articulation of the context in which a given item was originally seen. This view thus takes episodic remembering—i.e., recollection (Yonelinas, 2002: 446, see also Yonelinas, 2001)—to tap into type 2 metacognition only, taking feeling-based or type 1 metacognition to be involved exclusively in the production of the feeling of familiarity. No characteristic metacognitive feelings due to recollective processes, like the feeling of pastness, would be involved in remembering, therefore^14^.

While this approach has been influential, dissenting voices have emerged, in the form of a family of models on which recollection, too, involves type 1 metacognition (Whittlesea, 1997, 2003; Leboe and Whittlesea, 2002; Leboe-McGowan and Whittlesea, 2013), in particular (see also Kurilla and Westerman, 2008), has persuasively argued that the phenomenology of episodic recollection results from implicit detection and interpretation of possibly unconscious (or: subpersonal) cues^15^. Similar claims have been made by other researchers focused on metacognition, including (Koriat, 2007: 314; see also Nelson and Narens, 1990), or memory, including Jacoby et al. (1989), Kelley and Jacoby (1990); (see also Ansons and Leboe, 2011: 70), with the latter developing an attributionalist approach according to which the subjective experience of remembering is the result at the personal level of a subpersonal process of attribution to the past of detected procedural features like fluency^16^. In line with these models, we will defend a view of the phenomenology of remembering that sees it as arising specifically from type 1 metacognitive monitoring, but we will go beyond, in particular in arguing that it consists of a specific feeling – the feeling of pastness – distinct from the feeling of familiarity.

### Dokic’s Account

Only one philosopher—Dokic (2014)—has previously argued for a similar approach in any detail. On Dokic’s “two-tiered” account, remembering involves the production of both a first-order memory (the first tier, e.g., the imagistic representation of a scene that involves a birthday cake on a table surrounded by a bunch of people) and an associated feeling (the second tier, e.g., the feeling that this scene is an experience that I had in my personal past and from which my current image derives). The feeling in question is the result, at the personal level, of a cue detection and interpretation process occurring at the subpersonal level. Dokic sees these cues as being procedural in nature and thus treats the production of the feeling as being sensitive not to the *content* of the first-order memory but rather to features of the *process* that produces it (2012: 309; for similar claims, see Whittlesea, 1997: 219; Koriat, 2007: 298). This feeling, in turn, has its own content, that is the information it conveys. It is, Dokic argues, to the effect that the scene represented by the memory is presented as having occurred in the past experience of the remembering subject. Moreover, this content is, in Dokic’s view, different from the content the feeling initially possesses as a bodily feeling, for instance the procedural fluency. It is acquired through association due to regular co-occurrence. Feelings, such as the feeling of fluency, are indeed, in the first instance, conscious bodily conditions consisting in diffuse affective states (2012: 307). For instance, one can feel that reading a certain list of words is an easier task to carry out than reading another list, or that the answer to a certain question comes up in an easier way to one’s mind than the answer to another one. The occurrence of these conditions may covary with the occurrence of mental states, such as memory. Feelings of a certain type may thus become signs of states of a certain type, thereby acquiring a new content. Dokic maintains, in particular, that the feeling of fluency acquires the content that the represented event took place in the past due to its cooccurrence and consequent association with the state of episodic memory (2012: 308−9; s2014: 9−10)^17^.

This feeling belongs, Dokic argues, to the general category of *epistemic feelings*; more specifically, it belongs to the category of *feelings of knowing*. Epistemic feelings are metacognitive feelings with contents concerning the achievements and capacities of the subject’s cognitive system. The feeling of certainty, for example, might amount to a positive assessment of the answer that one has given to a question. The feeling of knowing (FOK) is the feeling that one has the ability to provide the correct answer to a certain epistemic task. It thus grounds a positive assessment of the subject about her own epistemic capacities relative to the task she has to carry out. For example, as one is asked to provide the name of the capital city of a certain country, one can feel that one is able to retrieve the name in question before one has actually managed to retrieve it. This much is standard. Dokic’s key move is to suggest that the FOK may occur not only *before* one has retrieved an item but also *while* the item is being retrieved and to characterize the phenomenology of episodic remembering as an *episodic feeling of knowing* (EFOK). If we understand him correctly, the notion is that as one is remembering, one feels that one’s mental state is epistemically well-grounded to the effect that one’s mental states derives in an appropriate way from one of one’s past experiences. In brief, one feels that one’s state is a state of episodic knowing.

There is much to like in Dokic’s account, and, in what follows, we will take three of his general claims on board. First, we grant that autonoetic phenomenology is a metacognitive feeling resulting from the subpersonal detection of certain cues by monitoring of the process that produces the first-order memories that it accompanies. Second, we agree with Dokic that the content of this pastness-related phenomenology is acquired. Finally, we accept his claim that epistemic feelings are bodily in nature. Our endorsement of these general claims notwithstanding, there are several major difficulties for the specific metacognitive feeling-based account in which Dokic embeds them.

First, while we agree with Dokic that features of the processing that produces memories contribute to the production of epistemic feelings and that the detection of these features is to an important extent content-insensitive, we disagree with his stronger claim that detection is entirely insensitive to content. The empirical results provided by Whittlesea’s experiments presented above demonstrate that the interpretation of the cues that trigger epistemic feelings is sensitive to the mental type (mnemonic, perceptual, etc.) that the context of processing of a first-order mental state attributes to the latter. Depending on the type of cognitive task a subject is required to carry out, he is likely to interpret one and the same cue differently, thus to experience different epistemic feelings. Now—so is our argument—since the mental type contributes to the content—for instance, if something is remembered rather than perceived, it is built into the content that the represented fact took place in the past—and since epistemic feelings are sensitive to the mental type, then one cannot say (*pace* Dokic) that epistemic feelings are insensitive to content. Second, we agree with Dokic on the bodily nature of feelings, that is: they are diffuse affective states and as such include a conscious sensory component that is about a certain bodily state (see section “The Phenomenality of the FOP: Accommodating the Inferentialist and the Embodied Views”). But we disagree with the claim that one can simply say that the initial bodily and the acquired contents of epistemic feelings occur at the personal level. There are robust empirical results demonstrating that the acquired content of feelings has to be substituted for the bodily states that feelings are about in order for feelings to play the role they play (Schacter and Singer, 1962; Jacoby and Whitehouse, 1989; Whittlesea et al., 1990; Whittlesea, 1997; Koriat, 2007: 314; see also Roediger and McDermott, 1995; Yonelinas, 2002: 464; Arango-Muñoz, 2011: 75). For instance, if fluency is made salient to a subject as the bodily content of his feeling of remembering, this feeling thereby ceases to be experienced as a feeling of remembering. Again, depending on the context of processing of a stimulus, that is the cognitive task the subjects carry out, the content of a feeling can be the bodily content (e.g., fluency), the pastness acquired content or a pleasantness content. Third, while we grant that the content of epistemic feelings is acquired, we deny that this fact should be described in terms of “co-occurrence,” “stable association” (Dokic, 2012: 308, 309), or cooccurrence “in normal context” with first-order episodic memory (Dokic, 2014: 10)^18^. Co-occurrence may explain why the feeling of pastness is associated with the represented scene, but it does not explain why pastness appears to the subject as a feature of the remembered scene—why it appears to be *attributed to* (*or: predicated of*) it. Finally, and most importantly, we argue that it is, at best, misleading to refer to autonoetic consciousness as an EFOK. Dokic borrows the notion of EFOK from the psychological literature. But we do not think he does full justice to the specific content given by that literature to the notion. Consider the view of the EFOK proposed by Souchay et al. (2007); (see also Souchay, 2007; Souchay and Moulin, 2009) and invoked by Dokic himself. Building on the model defended by Koriat (1993), on which what triggers the FOK with respect to a given item of information is brief and partial access to that information, Souchay et al. argue that this model applies to the EFOK, in particular, and that the partial information in question is then made of “the contextual information, feelings, and self-awareness captured in Tulving’s concept of autonoetic consciousness or the state of ‘remembering” (2007: 771). They argue therefore that the EFOK occurs when autonoetic consciousness in particular is partially accessed. If this view is right, then the EFOK cannot be *identified* with autonoetic consciousness simply because the former *results from* detection of the latter.

In light of this final point, we suggest that Dokic has failed to take into account a distinction between two kinds of epistemic feelings. On the one hand, there are feelings that concern the *capacities* of the cognitive system. The FOK and, for example, the tip-of-the-tongue state exemplify this kind, insofar as they tell the subject something about the mental states that his cognitive system is capable of producing. Note that the item of information to which a given instance of one of these feelings pertains is opaque to the subject, in the sense that the feeling informs him that he is capable of accessing the item before he has actually done so. On the other hand, there are feelings that concern the *achievements* of the cognitive system. The feeling of certainty and the feeling of familiarity exemplify this kind, insofar as they tell the subject something about the mental states that his cognitive system has already produced. The item of information to which a given instance of one of these feelings pertains is transparent to the subject, in the sense that the feeling informs him that he has successfully accessed the needed item. What Dokic misleadingly refers to as the “episodic feeling of knowing” belongs to the latter category, simply because it tells the subject something about a representation that has already been produced, namely, that it causally derives from the subject’s past experience of the represented event, while the FOK (EFOK included) belongs to the category of feelings relative to cognitive capacities. We emphasize that the fact that the FOK occurs before rather than while or after the relevant item is accessed (see Paynter et al., 2009) is not a contingent detail. It is precisely because the cognitive system has not yet accessed a given item that the subject needs to know whether it is worthwhile to continue to search for it; as the system succeeds in accessing the item, such knowledge becomes pointless. The motivational role of the FOK reinforces this point: as is widely acknowledged (Whittlesea, 1997: 220; De Sousa, 2009: 152), a core role of the FOK is to prompt the subject to continue to search for an item information, and this role, of course, cannot be played after the item has been accessed.

To sum up, we note that, if our critical discussion of Dokic is on the right track, then a satisfactory feeling-based account of episodic phenomenology will need to take the following points into account. First, the episodic phenomenology is an achievement-related epistemic feeling that differs from the FOK. Second, while the episodic phenomenology results from sensitivity to features of processing, it also displays a certain sensitivity to content. Third, the bodily conditions the detection of which triggers the episodic phenomenology have to be superseded by the acquired content in order for them to play their role. Fourth, the “pastness” involved in the episodic phenomenology is predicated of the represented scene, not merely associated with it. The metacognitive feeling-based account proposed in section “A New Metacognitive Feeling-Based Account” secures these four points.

## A New Metacognitive Feeling-Based Account

In this section, though Dokic’s account has proven to be unsatisfactory, we elaborate on its metacognitive two-tiered model of episodic remembering to promote a new metacognitive account. We argue that the phenomenology of remembering consists of a metacognitive feeling of pastness (FOP). To do so, we first tackle the issue of the relationship of a FOP to the memory with which it is associated; we then focus on the FOP itself, considering, in turn, its phenomenality and its content.

### The Relation of the FOP to First-Order Episodic Memory: Moderate Anti-separatism

The first issue is the relationship of a FOP to the first-order memory that it accompanies. Provided that the latter has a certain content, typically a represented scene, and that the FOP endows it with its phenomenology, what is the relationship between the content of the memory and the phenomenology, in particular the content of the accompanying feeling? Is the content of the feeling that the represented scene occurred in my past dependent on the content of the image that represents the scene, or is the former independent from the latter? And if the content of the memory and its phenomenology are linked by a dependence relation, in which direction does the relation run? Borrowing terms from Horgan and Tierson (2002) applied by Fernández (2017) to memory, we favor *moderate anti-separatism*, i.e., we claim that the intentional and phenomenological aspects are not completely separate. Let us explain. As our critical discussion of the approach in terms of non-metacognitive feelings is meant to show, the phenomenology of remembering is not sensitive to the contents of particular retrieved memories but rather to features of the processes that produce the latter. For this reason, we cannot endorse *strong anti-separatism*. But then why endorse anti-separatism at all, rather than endorsing *separatism*, as Dokic (2012) does? In our view, there are two dependence relations between content and phenomenology that prevent us from adopting separatism and suggest, instead, a qualified, moderate anti-separatist view: the FOP depends on the mental type of the content of the first-order memory and the content of the complete memory depends on the FOP.

In order to describe the first of these relations, it is necessary to emphasize there are actually two kinds of sensitivity to content. On the one hand, sensitivity can be to the components of the content and their articulation. For instance, on Fernández’ (2019) view of episodic memory content, the FOP derives from sensitivity to the causal component of the content. We have rejected this token-content sensitivity. But there may also be sensitivity to the mental type—e.g., perceptual, imaginary, or mnemonic type – of the content^19^. Now, the feeling-based metacognitive framework in psychology presented above provides empirical evidence that strongly support the existence of this second kind of sensitivity^20^. The lesson we draw from such experiments is that it is the context in which processing occurs that determines how the features of the processing (e.g., fluency) that are detected by metacognitive monitoring are interpreted. We thus endorse a *contextualist* view on which the type assigned by the context of processing to the first-order content processed determines the type of feeling liable to occur in this context, thus the own content of the feeling. As we saw in section “Dokic’s Metacognitive Feeling-Based Account,” Dokic is also in favor of the idea that epistemic feelings possess their content contingently. Our contextualist view, on which one and the same detected cue can trigger feelings with different contents, is somewhat similar to Dokic’s view. But whereas Dokic seems to favor the notion of a long-term contingency—since, on his view, it is a regular cooccurrence of certain feelings and certain mental states that makes the former represent the latter (2012: 308)—our contextualism favors short-term contingency instead, maintaining that each particular context determines the content of the occurrent feeling^21^. To sum up, a first component of our anti-separatism is that the content of the FOP depends on the mnemonic type that the context of processing attributes to the content of the first-order mental state to which FOP is associated.

The second dependence relation goes in the other direction: the content of the episodic memory depends on the FOP. We argue below for the intentionalist view that (at least some of) the phenomenal components of a mental state have content (Crane, 2001; Arango-Muñoz, 2014). On this view, as far as the FOP is concerned, its content contributes to the content of an episodic memory – the complete memory – by meshing with the content of the first-order memory that it accompanies. It is, we suggest, necessary to distinguish indeed between the content of a first-order memory—typically a scene—and the content of the complete memory, which includes the information that the represented scene has been experienced by the remembering subject at some moment of her past life, with at retrieval the content of the FOP being incorporated into the content of the complete memory. Overall, the type of the processed mental state determines the type of feeling liable to be triggered by it, and conversely, the feeling so triggered contributes to the content of the mental state whose processing triggered it in the first place. Our own anti-separatism thus combines moderate sensitivity of phenomenology to the content processed with dependence of the content of the complete memory on its phenomenology.

### The Phenomenality of the FOP: Accommodating the Inferentialist and the Embodied Views

Let us now focus on the FOP itself, beginning with its phenomenality. How to account for the “feeling” nature of the FOP? As far as we can see, two main options are available: the *inferentialist* view and the *embodied* view.

On the inferentialist view, the “phenomenal quality could be explained in terms of the idea that experience-based judgments are based on an inferential process that is not available to consciousness, and hence the outcome of that process has the phenomenal quality of a *direct, self-evident intuition*” (Koriat, 2007: 314, our emphasis; see also Koriat, 2007: 297–298 and 1993: 611)^22^. On Koriat’s view, the feeling nature of an epistemic feeling consists in the intuitive character of a certain metacognitive judgment. More precisely, the cognitive system is so wired that the detection of a certain cue in a certain context triggers an inferential operation. For instance, access to part of the answer to a question (e.g., the first letter of the name of a capital city) triggers an inference to the conclusion that the subject knows the answer as a whole (the full name of the city). It plays the role of a premise, the other being something like: “If part of the answer can be accessed, so can the whole answer.” But since this inferential process is subpersonal, as the conclusion of the inference occurs it is both endowed with the assertive strength provided by the inference from which it ensues and appears to the subject as having its assertive strength in and of itself, i.e., as a gut feeling, rather than as the output of an inference, which is what it actually is. Hence the feeling quality of the FOK would consist of this apparent gut feeling, and so it would go for epistemic feelings in general. There are a number of reasons in favor of the inferentialist view. It accommodates the fact that epistemic feelings come with varying degrees of intensity. If the detected cue is weak (for instance, only a small part of the answer is accessed), the conclusion of the inference will have a correspondingly weak level of assertiveness, e.g., one will feel a weak FOK. More importantly, inferentialism smoothly accommodates an important feature of the FOP. As one episodically remembers, one does not, strictly speaking, have a FOP that accompanies the remembered scene as if the former were juxtaposed to the latter^23^. Instead, *the scene itself* appears as a scene experienced in the past. In other words, pastness appears as one of the features of the represented scene, not as a separate mental state that co-occurs with the first-order memory. Thus, an attribution of pastness to the represented scene must occur at some point. Inferentialism gives us just that with the predication carried out by the conclusion that results from the inference^24^. As the inference concludes, pastness appears as intrinsic to the remembered scene.

On the (James-inspired) embodied view, the feeling quality does not consist of the apparent intuitiveness of a metacognitive judgment, but of the bodily nature of the vehicle of its content. “On this model, noetic feelings are first and foremost *bodily experiences*, i.e., experiences about bodily states. They are diffuse affective states registering internal physiological conditions and events” (Dokic, 2012: 307, our emphasis)^25^. For instance, as far as the FOP is concerned, the bodily event of a high level of fluency of processing brings about a feeling. This account would seem to encounter two difficulties, but both can be met. First, assigning a bodily content to the FOP might seem problematic. Indeed, as already noticed, if it is made salient to a subject that her feeling of pastness is due to a felt perceptual fluency, the bodily condition does not play the role of a metacognitive cue anymore and the FOP fades away. But we said above, drawing on empirical evidence, that a bodily feeling can change its content without ceasing to be a feeling. Depending on the context of its processing, one and the same bodily feeling vehicle can have different contents^26^. The conscious character of the bodily feeling is thus unproblematic provided it does not imply the conscious experience of a bodily condition as its content. Second, if one considers Dokic’s (2012) associationist version of the embodied view, one does not clearly see why, as we argued in section “Dokic’s Metacognitive Feeling-Based Account,” the bodily feeling appears as a feature of the represented scene rather than as a merely accompanying feeling. In other words, the embodied view has to explain why pastness is a phenomenological feature of the content of the first-order memory, rather than merely a juxtaposed mental state. Let us say at this stage that we endorse the embodied view and grant that the feeling quality of epistemic feelings is bodily^27^. But we also think that inferentialism says something true. On the inferentialist version of the embodied view we promote, the felt bodily change (e.g., a high level of fluency) triggers the inference whose outcome is the attribution of a feature (e.g., pastness) to the represented object (e.g., the scene represented by the first-order memory). As we said above, this is just what the phenomenology of episodic memories presents us with. Moreover, inference can also explain how the bodily feeling gets its metacognitive content in place of its original bodily content. It is the inference process that interprets the detected bodily change as meaning, for example, pastness. Overall, inferentialism turns out to be the good way of fleshing out the embodied view rather an opposite view.

### The Content of the FOP: Moderate Conceptualism

The last issue we tackle is the content of the FOP. We said that the FOP carries informational content about the scene represented by the first-order memory, and we have made it explicit in section “The Experience-of-Content Approach” which components are typically included in that content. This point raises several interesting though rarely tackled issues. In particular, is the content of the FOP non-conceptual, as its nature of feeling suggest? Or is it conceptual? If so, to what extent? Moreover, provided that the FOP is not only about the past, how do the different (possibly conceptual) components of its content relate to each other? Are they compositionally related, or are they simply clustered together, and how tightly are they related? For reasons of space, we make only some brief suggestions here.

Though in the minority, some authors have observed in recent years that epistemic feelings are likely to have various degrees of complexity^28^. Following up on a suggestion made by Arango-Muñoz (2014), we maintain that this complexity is a conceptual complexity of their content. For instance, framing effects are observed regarding epistemic feelings (Koriat et al., 2004: 654; Finn, 2008), which suggests a sensitivity of the latter to concepts and possibly their (at least partial) conceptual nature. A more straightforward argument is that the experienced content of some epistemic feelings requires involvement of concepts. For instance, it seems that a being can hardly feel that she knows or has forgotten something without mastering the concepts of knowledge and forgetting and the basic theory of mind in which they have sense. We also maintain that there is “a gradation among epistemic feelings: from non-conceptual to conceptual ones” (Arango-Muñoz, 2014: 199). For instance, the feeling of knowing (respectively, forgetting or error) should be contrasted with the feeling of certainty (respectively uncertainty). While they are all about the (respectively lack of) epistemic robustness of a piece of information, these feelings possess different degrees of conceptuality. Arguably, one can experience a feeling of certainty without possessing, let alone being conscious of the concept of certainty, as the behavior of an animal or of an infant can display. But as we have said, one can hardly experience a feeling of knowing without being conscious of the concept of knowledge and a fortiori possessing it. A certain enrichment of the human conceptual apparatus has to occur to go from the first to the second type of epistemic feelings. Our suggestion is that a similar enrichment is required to go beyond feelings of familiarity (FOF) and be able to experience the FOP. Note that even amongst researchers who endorse a feeling-based approach, some take it for granted that: “a feeling of familiarity is usually sufficient to make one feel one is remembering,” (Whittlesea, 1993: 1235)^29^. In line with the above suggestion, we argue instead that the FOP involves more conceptual complexity than the FOF. We said in section “Introducing the Feeling View of Episodic Phenomenology” that “pastness” is an umbrella term that covers various notions as its ingredients, namely:

• singular temporal addresses taking place in a unilinear, continuous, and irreversible time and endowed with a perspectival character (i.e., each address is a temporal position from which the whole line of time can be considered);
• the subjective character of the mentioned time (i.e., it is the temporal path of the existence of a subject, which requires the notion of a self as correspondingly temporally extended);
• a causal continuity (i.e., the before/after relation characteristic of the notion of time involved in the phenomenology of episodic recollection is a causal relation)^30^.

By contrast, the FOF does not involve many of the just mentioned ingredients. In particular, neither unilinearity, nor irreversibility and perspectivality are required, and accordingly, the notion of an extended self it involves is much simpler than the one involved in the FOP.

How should one endorse this conceptualist analysis, though? Taken without qualification, it is hardly tenable. Regarding epistemic feelings in general, if they could carry contents of whatever conceptual complexity, then the distinction between meta-representational and feeling-based metacognition would threat to crash down. The latter would differ from the former only by the type of vehicles of contents while its conceptual sophistication would be the same. But how could type-1 processes go fast and be intuitive if they involve conceptually rich contents? Regarding the FOP in particular, if all the mentioned conceptual ingredients were included into its content, there would be a serious threat of conceptual overload. All in all, so goes the objection, one should prefer an alternative view to conceptualism. For instance, inspired by Russell (2003: 151), one could suggest that the conceptual ingredients mentioned above are ingredients of a re-description of the feelings experienced during episodic recollection, not ingredients constitutive of their content. We think, yet, that there is an empirically well-grounded solution that preserves conceptualism under a moderate version that stirs a middle way between constitutive conceptualism and a re-descriptivist anti-conceptualist view.

Constitutive conceptualism assumes there is just one way for a concept to be a component of the content of a mental state, namely: by being part and parcel of it. But as developmental psychology suggests, cognitive reality is more complex. During the ontogenetic development of a cognitive system, certain concepts can be the causal conditions of the content of others without being included into them. According to Hoerl and McCormack, the concept of pastness involved in episodic recollection entertains just such a relation with the notions of self and causality (2001, 2007, 2011). Regarding the notion of self, the claim is that the perspectival and singularity-endowed notion of time proper to the FOP is developmentally dependent on a certain notion of self. For this notion of time to be in place, indeed, beforehand one has to be conscious of oneself as a temporally extended self who occupies a certain singular temporal perspective (the current one) among others within a unique series, some of which one has occupied and can take again through an imaginative exercise of decentering. Perspectivality and singularity features of time would thus be the causal effects of the antecedent acquisition of a specific notion of self, while the latter would not itself be a component of the notion of pastness (2001: 219)^31^. Regarding causality, similarly, the claim is that the unilinear and continuous notion of time proper to the FOP is developmentally dependent on a certain notion of systematic causal relations between the events that form the line of time. The acquisition of the ability to reason causally on how events relate to each other within time brings about the notion of a time formed of a unilinear continuous sequence of experienced events (2011: 454)^32^. In brief, the notions of self and causality would shape the notion of time in play in the FOP. Drawing on this developmental-psychology-inspired analysis, we suggest a moderate version of conceptualism on which the content of the FOP is formed of a concept of past only, but this specific concept is developmentally shaped by previously acquired notions of self and causality. This way we assign a moderate amount of conceptuality to the content of the FOP, while securing through the shaping relation the fact that it has the different components we assigned to it in section “The Experience-of-Content Approach”^33^. This analysis aligns neatly with our experience of the FOP. As the phrase “feeling of pastness” suggests, what is central in the content of the FOP is first and foremost the concept of a past temporal location. But if one is prompted to flesh out the content of the FOP, one is likely to end up assigning to it a subjective character and causal relations between singular experienced events. In our view, this amounts to making it explicit what has developmentally brought about the notion of pastness proper to FOP and reflects neatly the different ways the components of the content of FOP belong to the latter.

## Conclusion

To sum up, we have made a case for a metacognitive feeling-based account of the phenomenology of episodic recollection, which we have designated as a feeling of pastness. We have promoted a view that integrates opposite views – most of which had never been opposed so far – scattered across the philosophical and psychological literatures. Three tenets have turned out pivotal for our account. Firstly, instead of promoting either separatism or anti-separatism regarding the relationship of the feeling of pastness to the content of the first-order memory that it provides with its phenomenology, we have preferred a view that makes due room both to insensitivity to the components of the content of the first-order memory and to sensitivity to the mental mnemonic type of its content. Secondly, instead of endorsing either an inferentialist or an embodied stand regarding the feeling nature of the phenomenology of episodic recollection, we have preferred a view on which subpersonal inferences are required for the bodily state of feeling to be attributed to the content of the first-order memory. Thirdly, instead of favoring either conceptualism or anti-conceptualism regarding the content of the feeling of pastness, we have preferred a view that accommodates both the possibility for epistemic feelings to have a conceptual content and the requirement of conceptual paucity, thereby explaining why pastness has a central place within a content that has several components.

## Author Contributions

The main ideas and most of the text come from DP. KM wrote one section and provided a revision of the manuscript. AS contributed to the redaction of a section and carried out a clean-up of another section. All authors contributed to the article and approved the submitted version.

## Conflict of Interest

The authors declare that the research was conducted in the absence of any commercial or financial relationships that could be construed as a potential conflict of interest.

## Footnotes

1 “Feeling of pastness” refers to the phenomenology of episodic recollection in a way that aligns nicely with our account because it emphasizes the feeling nature of the phenomenology of remembering and features the temporal information it conveys as its central component. Note that though James (1890/1983: 570, 612) and Russell (1921/1997: 163) famously use that expression, it should be clear that we have our own distinct definition of it. Also note that the specific components of FOP are discussed in section “What Are the Components of the Feeling?” and that the meaning of “feeling” is discussed in depth in sections “Dokic’s Metacognitive Feeling-Based Account” and “A New Metacognitive Feeling-Based Account.”

2 We use “meta-representational” in line with common usage in metacognition studies, which usually restrict this term to explicit and judgment-based metacognition. Thus, it should be clear that as we rebut meta-representationalism about episodic phenomenology, we do *not* rebut the representational value of metacognitive feelings. Quite the contrary, our feeling-based account intends to make due right to the representational character of metacognition, though not in meta-representationalist terms.

3 See sections “Dokic’s Account” and “The Relation of the FOP to First-Order Episodic Memory: Moderate Anti-separatism” for arguments and details.

4 Nor do we claim that they are *sufficient* for the phenomenology of remembering—indeed, we suggest below that it includes a fourth component. As one of the reviewers rightly pointed it out to us, another potential component is actuality (see Mahr, 2020, for this view). Again, we only claim that our list is made of typical components.

5 Whether singularity is a feature of episodic remembering, as the vast majority of studies assume it, is in fact a matter of debate. It is not sure that strong singularism about episodic memory – namely the claim that for a mental state to qualify as an episodic memory, it has to be about a particular/singular event – is the view to prefer. It is not even sure that weak singularism – namely the claim that for a mental state to qualify as an episodic memory, it is typically about a particular/singular event – is correct (on this, see Andonovski, 2020). We do not want to pursue this issue here.

6 Matthen (2010), Michaelian (2016), too, endorse non-metacognitive feeling accounts.

7 Fernández treats episodic memory as being propositional in character, but this feature of his approach will play no role in our assessment of it.

8 Fernández distinguishes two components in the temporal phenomenology of remembering: the awareness of an objective fact as obtaining in the past and the awareness of what it was like to perceptually experience that fact (APE) (2019: 87). Since he offers the same intentionalist account of both components, we will, for the sake of simplicity, consider only PAST in what follows.

9 In this section we present these findings to show that they render Fernandez’s strong anti-separatism implausible. They will also be pivotal to show that Dokic’ separatism is implausible (section “Dokic’s Account”) and that our moderate anti-separatism should be preferred (section “The Relation of the FOP to First-Order Episodic Memory: Moderate Anti-separatism”).

10 “Turning to the relationship between pleasantness and recognition ratings, it is clear that they were influenced by the same factors, and to about the same degree” (1993: 1246).

11 Because fluency-detection is considered as a chief heuristic in the literature on remembering, we will focus on it throughout the article. But there are other procedural features (like the ease of generation of further contextual details, for instance) that can play the same role. On this, see Whittlesea (1997: 219), Whittlesea and Leboe (2000).

12 We use “conscious” and “personal” equivalently, and refer thereby to “consciousness” in a wide sense that covers both its phenomenal and intentional features.

13 On its technical use in the mentioned experimentalist literature, the expression “recognition memory” refers to the mental states that prompts subjects to give an “old” response as they have to decide whether or not they have already been presented with an item. In addition to recollection in recognition memory, the literature distinguishes free recollection (or: free recall). But since the same analysis is proposed of the latter as of recollection as it is involved in recognition memory, considering this form of memory will suffice.

14 Note that the strong difference of nature posited by the standard dual processing approach between familiarity and recollection does not imply that it would exclude any functional relationship between them. For instance, some consider that an occurrence of familiarity triggers the search for contextual information through the recollection of specifics (Hintzman and Curran, 1994; Koriat and Levy-Sandot, 2001). Thanks to an reviewer for prompting this clarification. Also note that we do not exclude that a feeling of familiarity overlaps with recollection. What we contest is that this is a feeling due to recollective process, on the one hand, and on the other hand, that familiarity provides remembering with its characteristic phenomenology.

15 “(…) very little attention has been paid to (…) the ways in which attributional processes may contribute to a subjective sense of recollection in recognition memory.” (Kurilla and Westerman, 2011: 80).

16 Whittlesea himself recognizes the similarity of his view to attributionalism (1997: 213).

17 As suggested by Dokic (2012), this high availability of non-sensory bodily affection to acquire various contents different from their original bodily content is one of their interesting differences with respect to both emotional and sensory bodily affections. We do not pursue this point here.

18 Arango-Muñoz holds a similar view as he says that metacognitive feelings “are embodied signals that have been recruited to represent internal processes.” Arango-Muñoz (2018: 2).

19 One could also elaborate on this second kind of sensitivity in terms of a sensitivity to the cognitive mode under which the content of the first-order mental state is conscious. The resulting account, however, would be slightly different from the one favored here.

20 See section “The Experience-of-Content Approach” and “Dokic’s Account.” By way of a further empirical instance: in a series of experiments, Whittlesea and Leboe (2000) show that the occurrence of the same cues (fluency, generation, and resemblance) is recruited to perform cognitive operations as different as identifying a stimulus and remembering a previous experience of a stimulus. Depending on the questions posed to subjects, the detected cues will be interpreted differently; that is, depending on the mental type determined by the context in which the subject is prompted to process a content, he will experience a feeling of familiarity or a feeling of a successful identification (2000: 99).

21 See for instance Whittlesea (1997: 220). Some go as far as saying that one and the same cue can trigger feelings with contrary contents depending on the task context. For instance, fluency could trigger a feeling of familiarity as well as a feeling of novelty (Unkelbach, 2006). But see Olds and Westerman (2012) for a critical discussion of these results.

22 The claim that the feelings that result from the metacognitive monitoring are derived through an inference is widespread in the empirical literature on feeling-based metacognition (Jacoby et al., 1989; Whittlesea, 1993, 1997; Koriat, 2000; Kurilla and Westerman, 2011).

23 See above and below our criticism of Dokic’s account on this point.

24 Note that, just after the passage quoted above, Koriat insists on the “nice convergence” there is between his feeling-based metacognitive view and the attributionalist view promoted by Kelley and Jacoby (1990). The same claim is made in Koriat (1993: 611).

25 See Damasio (1999), Prinz (2004) for prominent advocates of this view.

26 See our discussion of Dokic’s account on this point in section “Dokic’s Account.”

27 For a review of some empirical arguments, see Arango-Muñoz (2014).

28 Dokic (2012: 308) suggests a distinction between basic and non-basic epistemic feelings.

29 Whittlesea also speaks of the “feeling of pastness” (1997: 241). But there is no indication he intends to distinguish it from the feeling of familiarity.

30 McCormack and Hoerl (1999), Hoerl and McCormack (2001, 2011), McCormack and Hoerl (2007).

31 “Self-consciousness has a substantive *causal* role in the emergence of episodic memory rather than being a necessary component of episodic recollection” (210).

32 “We argue that the ability to engage in this form of reasoning is central to possessing the concept of the past that is used in episodic memory and that children do not possess this concept until they are capable of engaging in this type of reasoning” (2001: 218).

33 As pointed out by a referee, an important question here is whether reducing the number of the components of the FOP suffices to reduce its conceptual complexity. After all, one could say, the developmental shaping relation we invoke can propagate into the FOP the complexity of the components that it does not include. Our tentative reply is that for a given set of concepts, it is less demanding to think of just one of them rather of the whole conjunction of them, even though the former has been complexified by the latter. As we suggest at the beginning of section “The Content of the FOP: Moderate Conceptualism,” pursuing this point is beyond the scope of the present article.
